# Combined forced diuresis and late acquisition on [^68^Ga]Ga-PSMA-11 PET/CT for biochemical recurrent prostate cancer: a clinical practice-oriented study

**DOI:** 10.1007/s00330-023-09516-0

**Published:** 2023-03-09

**Authors:** Matteo Bauckneht, Alberto Miceli, Alessio Signori, Domenico Albano, Selene Capitanio, Roberta Piva, Riccardo Laudicella, Annalisa Franchini, Francesca D’Amico, Mattia Riondato, Silvia Chiola, Cecilia Marini, Giuseppe Fornarini, Antonio Scarale, Alfredo Muni, Francesco Bertagna, Irene A. Burger, Gianmario Sambuceti, Silvia Morbelli

**Affiliations:** 1 Nuclear Medicine, IRCCS Ospedale Policlinico San Martino, Genoa, Italy; 2 Department of Health Sciences (DISSAL), University of Genova, Genoa, Italy; 3 Nuclear Medicine, University of Brescia and ASST Spedali Civili Brescia, Brescia, Italy; 4 Nuclear Medicine ASST, Grande Ospedale Metropolitano Niguarda, Milan, Italy; 5 Nuclear Medicine Unit, Azienda Ospedaliera SS. Antonio E Biagio E Cesare Arrigo, Alessandria, Italy; 6 Nuclear Medicine, Cantonal Hospital Baden, Baden, Switzerland; 7 Nuclear Medicine, University Hospital Zurich, University of Zurich, Zurich, Switzerland; 8 Nuclear Medicine Unit, Department of Biomedical and Dental Sciences and Morpho-Functional Imaging, University of Messina, Messina, Italy; 9 CNR Institute of Molecular Bioimaging and Physiology, Milan, Italy; 10 Medical Oncology Unit 1, IRCCS Ospedale Policlinico San Martino, Genoa, Italy

**Keywords:** Prostate cancer, PSMA, PET/CT, Biochemical recurrence, Diagnostic accuracy

## Abstract

**Objectives:**

Increased detection of prostate cancer (PCa) recurrences using [^68^Ga]Ga-PSMA-11 PET/CT has been reported by adding forced diuresis or late-phase imaging to the standard protocol. However, the combination of these procedures in the clinical setting is still not standardized.

**Methods:**

One hundred prospectively recruited biochemical recurrent PCa patients were restaged with dual-phase [^68^Ga]Ga-PSMA-11 PET/CT from September 2020 to October 2021. All patients received a standard scan (60 min), followed by diuretics (140 min) and a late-phase abdominopelvic scan (180 min). PET readers with low (*n* = 2), intermediate (*n* = 2), or high (*n* = 2) experience rated (i) standard and (ii) standard + forced diuresis late-phase images in a stepwise fashion according to E-PSMA guidelines, scoring their level of confidence. Study endpoints were (i) accuracy against a composite reference standard, (ii) reader’s confidence level, and (iii) interobserver agreement.

**Results:**

Forced diuresis late-phase imaging increased the reader’s confidence category for local and nodal restaging (both *p* < 0.0001), and the interobserver agreement in identifying nodal recurrences (from moderate to substantial, *p* < 0.01). However, it significantly increased diagnostic accuracy exclusively for local uptakes rated by low-experienced readers (from 76.5 to 84%, *p* = 0.05) and for nodal uptakes rated as uncertain at standard imaging (from 68.1 to 78.5%, *p* < 0.05). In this framework, SUVmax kinetics resulted in an independent predictor of PCa recurrence compared to standard metrics, potentially guiding the dual-phase PET/CT interpretation.

**Conclusions:**

The present results do not support the systematic combination of forced diuresis and late-phase imaging in the clinical setting, but allow the identification of patients-, lesions-, and reader-based scenarios that might benefit from it.

**Key Points:**

*• Increased detection of prostate cancer recurrences has been reported by adding diuretics administration or an additional late abdominopelvic scan to the standard [* ^*68*^ *Ga]Ga-PSMA-11 PET/CT procedure.*

*• We verified the added value of combined forced diuresis and delayed imaging, showing that this protocol only slightly increases the diagnostic accuracy of [* ^*68*^ *Ga]Ga-PSMA-11 PET/CT, thus not justifying its systematic use in clinics.*

*• However, it can be helpful in specific clinical scenarios, e.g., when PET/CT is reported by low-experienced readers. Moreover, it increased the reader's confidence and the agreement among observers.*

**Supplementary Information:**

The online version contains supplementary material available at 10.1007/s00330-023-09516-0.

## Introduction

In the last decade, prostate-specific membrane antigen (PSMA) positron emission tomography/computed tomography (PET/CT) became the first-choice imaging for the restaging of biochemical recurrence (BCR) of prostate cancer (PCa) [1]. Among the numerous ligands developed for PSMA-targeted imaging [2], [^68^Ga]Ga-PSMA-11 has shown great promise, having high diagnostic accuracy [3–5] and impact on clinical management [6], particularly in the presence of low prostate-specific antigen (PSA) levels [7].

However, the identification of sites of recurrence with [^68^Ga]Ga-PSMA-11 is partly limited by the tracer’s urinary excretion, which may reduce the accuracy when the residual disease is in the prostate bed or peri-ureteral nodes [8] thus resulting in inconsistent reporting, especially by lower expert readers [9].

In the latest version of the joint EANM/SNMMI [^68^Ga]Ga-PSMA-11 PET/CT procedural guidelines [10], the administration of furosemide (20 mg) or additional delayed abdominopelvic imaging is recommended as optional procedures able to improve the visualization of peri-ureteral and peri-bladder tissues [11]. However, protocols for forced diuresis, delayed imaging, and, more importantly, their combination are still not standardized in the clinical setting and their clinical added value still needs to be clarified. Given the practical implications of implementing these demanding procedures in clinical practice, it would be highly relevant to disclose specific clinical scenarios that might benefit from it.

On these bases, we systematically added forced diuresis and late abdominopelvic acquisition to the standard [^68^Ga]Ga-PSMA-11 PET/CT protocol, in order to verify its added value in terms of (i) diagnostic accuracy, (ii) readers’ confidence, and (iii) interobserver agreement compared to the standard procedure among PET readers with various levels of expertise.

## Patients and methods

### Patients and imaging procedures

**Fig. 1:**
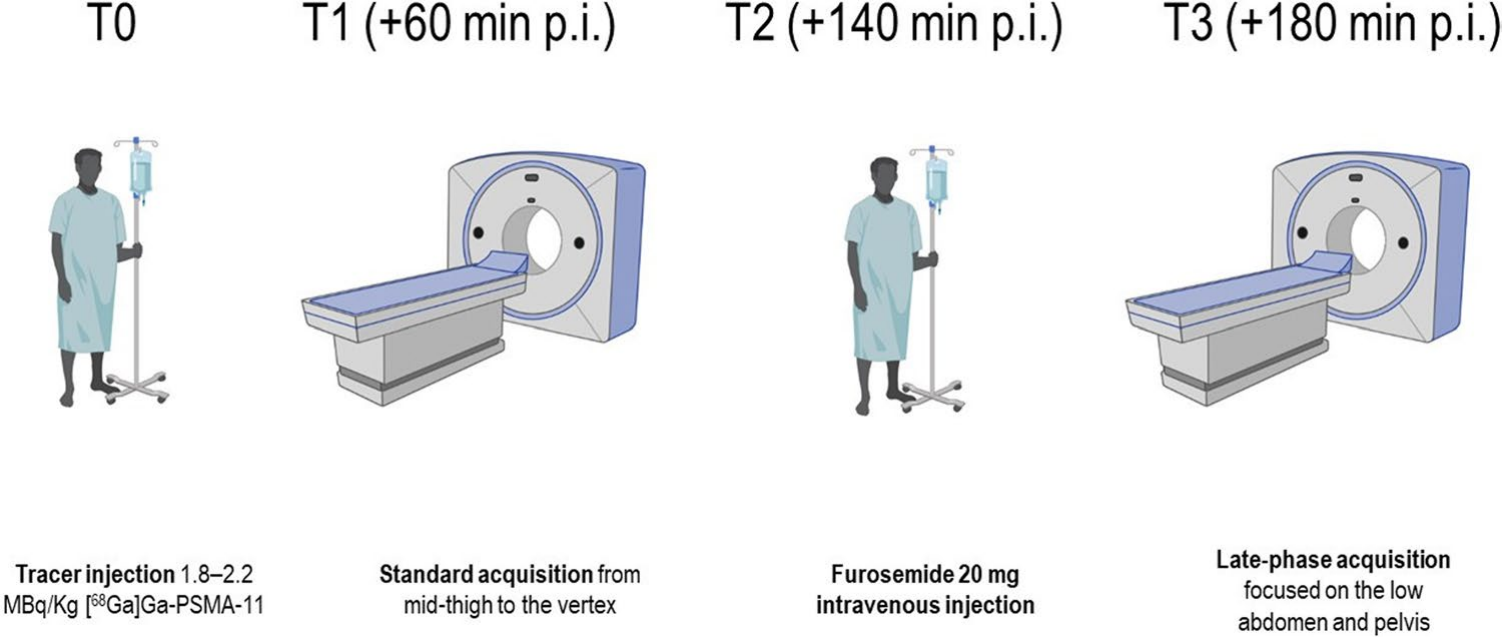
Illustrative representation of the imaging protocol of the study. Patients received 2 MBq/kg of [^68^Ga]Ga-PSMA-11 followed by standard 60 min p.i. imaging. At 140 min p.i., all patients received 20 mg of Furosemide intravenously, followed by an acquisition of the pelvis and lower abdomen at 180 min p.i

BCR patients were prospectively enrolled in the study between September 2020 and October 2021. Patients were enrolled according to the following inclusion criteria: (i) age ≥ 18 years old; (ii) histologically proven PCa previously treated with definitive therapy; (iii) BCR defined as a PSA increase ≥ 2.0 ng/mL above the nadir value after radiotherapy or a PSA rise of ≥ 0.2 ng/mL after prostatectomy; (iv) referred to [^68^Ga]Ga-PSMA-11 PET/CT for restaging. The sole exclusion criterion was the clinical contraindication to furosemide administration. The local ethical committee approved the study (Regional Ethical Committee of Liguria-registration number 343/2019). Informed consent was obtained before the PET/CT scan from all individual participants included in the study. All patients received 1.8–2.2 MBq/kg of [^68^Ga]Ga-PSMA-11 intravenously according to international guidelines [10]. Patients were orally hydrated and were asked to void before imaging acquisition. PET/CT scanners using either Hirez-Biograph 16 (Siemens Medical Solutions) or Biograph mCT Flow (Siemens Medical Solutions) were used to perform a PET acquisition at 60 min p.i. from the skull vertex to the upper thighs (termed “standard scan”) in the three-dimensional mode. At 140 min p.i., all patients received furosemide 20 mg intravenously followed by an abdominopelvic acquisition (at 180 min p.i., termed “forced diuresis late-phase scan”). An illustrative summary of the study protocol is represented in Fig. 1.

### Imaging interpretation

Six PET readers were classified as having low (< 30 prior studies, *n* = 2), intermediate (30–300 studies, *n* = 2), or high level of experience (> 300 studies, *n* = 2) for [^68^Ga]Ga-PSMA-11 PET/CT reporting [9]. All readers, blinded to reference standard, rated (i) standard acquisitions alone and (ii) standard + forced diuresis late acquisitions in a stepwise fashion. A washout period of 3 months was given between the two rounds of reporting. Readers had full access to patient history, PSA levels, PSA kinetics, and the pre-test probability as assessed by a validated prediction nomogram [12, 13]. The following patient data were also disclosed to each observer before image interpretation: age, weight, injected dose, initial PSA level, type of radical treatment (surgery/radiotherapy), biopsy and histopathological features including Gleason Score, International Society of Urological Pathology prostate grade group, eventual adjuvant therapy, PSA nadir.

Readers were asked to report [^68^Ga]Ga-PSMA-11 PET/CT images according to EANM guidelines [14], considering the common pitfalls [15] and using the standardized nomenclature for lymph node regions [16]. For each round of reporting, readers were asked to visually review imaging results to record the study’s general, local, and nodal impression (positive/negative/equivocal). The following data were asked to be recorded for each suspected local (T), nodal (N), or distant recurrence (M): (i) highest maximum standardized uptake value (SUVmax) for the most representative target region; (ii) PSMA uptake compared to the background in the blood, liver, and salivary glands rated on a visual scale of 0–3 (PSMA visual score [14]); (iii) personal confidence to quantify the likelihood of the presence of PCa (from 1 = certain benign to 5 = certain pathological); (iv) PSMA reporting and data systems (PSMA-RADS) according to current guidelines [14] (see also the Supplementary Materials).

### Standard reference definition

When present, post-surgical histopathology was considered the reference standard. Clinical, biochemical, and radiological follow-up served as the composite reference standard in the remaining cases as previously described [17]. Lesions were confirmed by the change in size, disappearance or appearance on follow-up imaging, or PSA drop of ≥ 50% after focal salvage therapy. The local investigators interpreted the composite reference standard after reviewing follow-up information. [^68^Ga]Ga-PSMA-11 PET/CT positive findings were validated as true- or false-positive on a regional basis.

When present, post-surgical histopathology was considered the reference standard. Clinical, biochemical, and radiological follow-up served as the composite reference standard in the remaining cases as previously described [17]. Lesions were confirmed by the change in size, disappearance or appearance on follow-up imaging, or PSA drop of ≥ 50% after focal salvage therapy. The local investigators interpreted the composite reference standard after reviewing follow-up information. [^68^Ga]Ga-PSMA-11 PET/CT positive findings were validated as true- or false-positive on a regional basis.

### Statistical analyses

Continuous data are expressed as mean ± standard deviation. For each PET reader general, local and nodal true positives (TPs), false positives (FPs), true negatives (TNs), false negatives (FNs), sensitivity, and specificity were calculated using the above-described composite reference standard. Equivocal findings were considered not consistent with the reference standard. Diagnostic accuracy was measured according to the formula (TP/FN)/(FP/TN). Agreements between reader’s reports and the reference standard were measured using Cohen’s kappa analyses, while differences in diagnostic accuracy were calculated using the chi-square test to compare proportions. Differences between early and late reports were assessed by a generalized estimating equation (GEE) model to consider multiple observations for each patient. Semiquantitative imaging parameters were included in uni- and multivariable models using a GEE logistic regression model for clustered data, estimating odds ratios, and their 95% confidence intervals (95% CI). Factors with a *p* < 0.10 at the univariable analysis were included in the multivariable analyses. The area under the receiver-operating-characteristic curve (AUC) and 95% CI were calculated using an approach for clustered data [18] and were used to evaluate the ability of key parameters to determine diagnoses. Cutoffs for key parameters were inferred based on the Youden index [19]. Reader’s levels of confidence for PCa recurrence were grouped as follows: certain (level of confidence = 1 or 5), intermediate (level of confidence = 2 or 4), and unknown (level of confidence = 3). Confidence classes were reported as the median and interquartile range according to the degree of the reader’s experience. Differences between standard acquisition and standard + forced diuresis late acquisition were assessed by using a Wilcoxon signed-rank test. The same analysis was performed considering PSMA-RADS classes, grouped as follows: certain (PSMA-RADS = 1 or 5), intermediate (PSMA-RADS = 2 or 4), and unknown (PSMA-RADS = 3). Agreement between observers was measured as the proportion of consistent reader impressions. Cohen’s kappa (2 raters) and Fleiss kappa (> 2 raters) were calculated to assess the interobserver agreement [20]. Interpretation of kappa was performed as previously described [21]: 0.0, poor; 0.0–0.20, slight; 0.21–0.40, fair; 0.41–0.60, moderate; 0.61–0.80, substantial; 0.81–1.00, almost-perfect reproducibility. Statistical analyses were performed using MedCalc v19.4 (MedCalc Software).

## Results

### Patients’ characteristics and reference standard

From 112 initially selected patients, 12 were excluded for a contraindication to furosemide administration (1/12 allergy to sulfonamides; 11/12 hypotension). The clinical characteristics of included patients are summarized in Table 1. The mean PSA at the time of imaging was 1.6 ± 3.2 ng/mL (range 0.18–27.2 ng/mL). Histologic verification (*n* = 3), PSA response after external-beam radiation (*n* = 44), and imaging follow-up (*n* = 26) served as the reference standard in most cases. In the remaining patients (*n* = 27), PET/CT images were analyzed in a joint reading session by consensus of 2 expert readers [9], aware of all clinical data and validated through a multidisciplinary discussion. According to the standard of reference, [^68^Ga]Ga-PSMA-11 PET/CT scans were negative in 38/100 patients (38%). Local, nodal, and distant recurrences were present in 27, 36, and 14 patients, respectively.

**Table 1:** Patients’ characteristics

Age (years)	65.9 (50–85)
initial PSA (ng/mL)	15.6 (2.6–173)
Primary diagnosis validated by histopathology	100/100 (100%)
ISUP grade at diagnosis	
*ISUP grade 1*	15 (15%)
*ISUP grade 2*	25 (25%)
*ISUP grade 3*	27 (27%)
*ISUP grade 4*	16 (16%)
*ISUP grade 5*	17 (17%)
Initial treatment	
*Surgery*	82 (77)
*Radiotherapy* ± *ADT*	17 (17%)
*Adiuvant radiotherapy*	16 (16%)
*Adiuvant ADT*	30 (30%)
PSA nadir (ng/mL)	0.16 (0.00–4.15)
Salvage radiotherapy	23/82 (28%)
Setting of PET/CT restaging	
*First time BCR*	42 (42%)
*BCR after salvage radiotherapy*	35 (35%)
*Biochemical persistence after surgery*	9 (9%)
*Biochemical progression during ADT*	14 (14%)
PSA and PSA kinetics at BCR	
*PSA (ng/mL)*	1.6 (0.18–27.2)
*PSAdt (months)*	9.7 (0.2–53)
*PSAvel (ng/mL/year)*	1.9 (0.1–53.1)
ADT at PET/CT	18 (18%)
Interval between initial treatment and BCR (months)	45.2 (1–190)
PET/CT pretest probability*	48.8% (4–96%)

*PSA* prostate-specific antigen, *ISUP* International Society of Urological Pathology, *ADT* androgen deprivation therapy, *PET/CT* positron emission tomography/computed tomography, *BCR* biochemical recurrence, *PSAdt* prostate-specific antigen doubling time, *PSAvel* prostate-specific antigen velocity; * defined according to [12, 13]

### Impact of forced diuresis late-phase imaging on diagnostic accuracy

At the per-patient analysis, adding the forced diuresis late-phase imaging, the overall diagnostic accuracy slightly (though not significantly) increased from 75.6% (95% CI: 0.48–0.60, kappa = 0.54) to 79.3% (95% CI: 0.52–0.64, kappa = 0.58) (*p* = 0.12). Further details about diagnostic accuracy, including M-status, are reported in the Supplementary Materials. At the per-lesion analysis, forced diuresis late-phase imaging significantly increased nodal accuracy from 78.3% (95% CI: 0.45–0.58, kappa = 0.51) to 84.7% (95% CI: 0.58–0.70, kappa = 0.64) (*p* < 0.01), while its effect did not reach significance for local accuracy, which increased from 78% (95% CI: 0.36–0.50, kappa = 0.43) to 81.3% (95% CI: 0.41–0.55, kappa = 0.48) (*p* = 0.15). However, grouping readers according to their previous experience, it significantly improved the local diagnostic accuracy of low-experienced readers (Table 2).

**Table 2:** Changes in diagnostic accuracy according to the reader’s previous experience

	Standard			Standard + forced diuretic late-phase			*p*
	Accuracy (%)	*Kappa*	*95% CI*	Accuracy (%)	*Kappa*	*95% CI*	
**General accuracy**							
*Low experience*	74.5%	0.52	0.42–0.62	80%	0.61	0.51–0.71	0.19
*Intermediate experience*	71.5%	0.48	0.39–0.58	74%	0.48	0.37–0.59	0.57
*High experience*	81%	0.62	0.52–0.72	84%	0.65	0.55–0.75	0.43
**Local accuracy**							
*Low experience*	76.5%	0.40	0.29–0.52	84%	0.56	0.44–0.69	**0.05**
*Intermediate experience*	77.5%	0.41	0.29–0.53	76.5%	0.37	0.25–0.50	0.81
*High experience*	80%	0.46	0.35–0.58	83%	0.51	0.39–0.63	0.44
**Nodal accuracy**							
*Low experience*	78%	0.52	0.42–0.63	83.5%	0.63	0.52–0.73	0.16
*Intermediate experience*	77.5%	0.50	0.38–0.61	84%	0.63	0.52–0.74	0.09
*High experience*	79.5%	0.52	0.42–0.63	86.5%	0.66	0.56–0.77	0.06

### Impact of forced diuresis late-phase imaging on the reader's confidence level

The mean overall confidence category increased after reading standard + forced diuresis late-phase scans compared to standard acquisition regarding both the local and nodal status (both *p* < 0.0001, Table 3). The increase in reader’s confidence level was confirmed for both local and nodal lesions when readers were grouped according to their previous experience (Table 3). In both cases, readers with lower levels of expertise took the higher advantage of forced diuresis late-phase imaging, showing higher numbers of positive differences compared to the standard scan.

**Table 3:** Changes in confidence levels according to the reader’s previous experience

			Overall	Low experience	Intermediate experience	High experience
**Local status**	**Confidence level**	Mean confidence at standard imaging	2.48 [IQR. 2.0–3.0]	2.47 [IQR. 2.0–3.0]	2.47 [IQR. 2.0–3.0]	2.51 [IQR. 2.0–3.0]
		Mean confidence at standard + forced diuresis	2.72 [IQR. 3.0–3.0]	2.82 [IQR. 3.0–3.0]	2.68 [IQR. 3.0–3.0]	2.66 [IQR. 3.0–3.0]
		Number of positive differences	140	59	42	39
		Number of negative differences	25	6	11	8
		*p* value	** < 0.0001**	** < 0.0001**	** < 0.0001**	**0.0006**
	**PSMA RADS**	Mean confidence at standard imaging	2.23 [IQR. 2.0–3.0]	2.08 [IQR. 2.0–3.0]	2.32 [IQR. 2.0–3.0]	2.28 [IQR. 2.0–3.0]
		Mean confidence at standard + forced diuresis	2.32 [IQR. 2.0–3.0]	2.55 [IQR. 2.0–3.0]	2.25 [IQR. 2.0–3.0]	2.23 [IQR. 2.0–3.0]
		Number of positive differences	34	17	8	9
		Number of negative differences	15	1	8	6
		*p* value	0.0561	**0.0002**	0.8603	0.8904
**Nodal status**	**Confidence level**	Mean confidence at standard imaging	2.56 [IQR. 2.0–3.0]	2.58 [IQR. 2.0–3.0]	2.52 [IQR. 2.0–3.0]	2.58 [IQR. 2.0–3.0]
		Mean confidence at standard + forced diuresis	2.78 [IQR. 3.0–3.0]	2.83 [IQR. 3.0–3.0]	2.75 [IQR. 3.0–3.0]	2.77 [IQR. 3.0–3.0]
		Number of positive differences	123	43	44	36
		Number of negative differences	18	6	7	5
		*p* value	** < 0.0001**	** < 0.0001**	** < 0.0001**	** < 0.0001**
	**PSMA RADS**	Mean confidence at standard imaging	2.11 [IQR. 1.0–3.0]	2.04 [IQR. 1.0–3.0]	2.22 [IQR. 1.0–3.0]	2.11 [IQR. 1.0–3.0]
		Mean confidence at standard + forced diuresis	2.52 [IQR. 2.0–3.0]	2.57 [IQR. 2.5–3.0]	2.61 [IQR. 3.0–3.0]	2.42 [IQR. 1.0–3.0]
		Number of positive differences	40	16	12	12
		Number of negative differences	6	2	3	1
		*p* value	** < 0.0001**	**0.0019**	**0.0103**	**0.0081**

The analysis of PSMA-RADS changes did not reproduce the same findings. Regarding the local status, the mean PSMA-RADS category slightly (though not significantly) increased after reading standard + forced diuresis late scans compared to standard acquisition. Only low-experienced readers significantly increased the PSMA-RADS scores between the two reporting rounds (Table 3). By contrast, nodal PSMA-RADS scores significantly increased after reading standard + forced diuresis late scans (*p* < 0.0001), regardless previous experience (Table 3).

PSMA-RADS system showed less sensitivity in tracking the increase in reader’s confidence compared to confidence categories, as PSMA-RADS classes increased from standard to standard + forced diuresis late-phase images in 34 and 40 cases while confidence classes increased in 140 and 123 cases for local and nodal status, respectively.

### Impact of forced diuresis late-phase imaging on diagnostic accuracy according to the reader's confidence level

Forced diuresis late-phase imaging significantly improved the diagnostic accuracy exclusively in the case of nodal uptakes rated with low-to-intermediate confidence at standard imaging (Table 4). By contrast, no advantages in diagnostic accuracy were observed for nodal uptakes rated with high confidence at standard imaging and for local uptakes, regardless of the confidence level (Table 4).

**Table 4:** Changes in diagnostic accuracy according to the reader’s level of confidence

	Standard			Standard + forced diuretic late-phase			*p*
	Accuracy (%)	*Kappa*	*95% CI*	Accuracy (%)	*Kappa*	*95% CI*	
**Local accuracy**							
*Low/intermediate confidence*	70.4%	0.43	0.32–0.53	73.4%	0.46	0.35–0.57	0.47
*High confidence*	87.9%	0.62	0.52–0.72	88.2%	0.64	0.54–0.74	0.89
**Nodal accuracy**							
*Low/intermediate confidence*	68.1%	0.38	0.26–0.49	78.5%	0.57	0.45–0.68	** < 0.05**
*High confidence*	90.2%	0.75	0.69–0.82	91.4%	0.79	0.72–0.85	0.55

### Standard and forced diuresis late-phase imaging semiquantitative parameters in the prediction of true positive T and N lesions

**Fig. 2:**
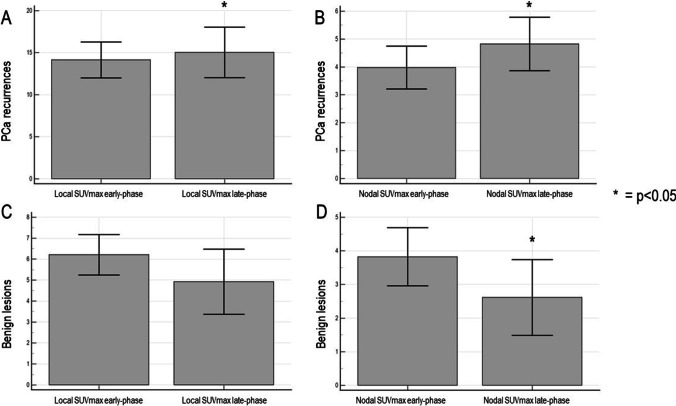
Divergent kinetics of SUVmax in local and nodal metastases vs. benign lesions. (**A**, **B**) The increase in SUVmax local and nodal metastases, (**C**, **D**) the opposite kinetics by local and nodal uptakes resulting in benign according to the reference standard

Increased [^68^Ga]Ga-PSMA-11 uptake at late imaging characterized local and nodal PCa recurrences. Indeed, SUVmax significantly increased from standard to forced diuresis late-phase images in local (from 3.76 ± 8.7 to 4.24 ± 10.9, *p* = 0.011 [GEE model], Fig. 2(A)) and nodal recurrences (from 3.98 ± 8.7 to 4.82 ± 10.8 *p* = 0.019 [GEE model], Fig. 2(B)). By contrast, SUVmax slightly decreased without reaching the significance in local benign lesions (from 6.12 ± 3.8 to 4.85 ± 6.2, *p* = 0.18 [GEE model], Fig. 2(C)) and significantly decreased in nodal benign lesions (from 3.82 ± 3.1 to 2.61 ± 4.1 *p* = 0.019 [GEE model], Fig. 2(D)).

SUVmax variation from standard to late forced acquisition imaging (ΔSUVmax) was included in uni- and multivariable logistic regression model containing standard imaging semiquantitative parameters indicated by the E-PSMA reporting guidelines (SUVmax, PSMA score, PSMA-RADS) [14] in the prediction of PCa recurrences. Table 5 shows that ΔSUVmax significantly and independently predicted the presence of local and nodal PCa recurrences in addition to the remaining semiquantitative imaging parameters.

**Table 5:** Uni- and multivariable logistic regression model for the prediction of T- and N-true positive lesions

	Univariable			Multivariable		
	*OR*	*95% CI*	*p*	*OR*	*95% CI*	*p*
**T-lesions semiquantitative parameters**						
SUVmax at standard imaging	1.20	1.14–1.26	** < 0.001**	1.27	1.13–1.43	** < 0.001**
PSMA score at standard imaging	3.36	2.53–4.47	** < 0.001**	1.56	0.81–3.01	0.19
PSMA RADS at standard imaging	1.61	1.43–1.82	** < 0.001**	1.01	0.81–1.24	0.95
deltaSUVmax	1.04	1.01–1.06	**0.025**	1.16	1.10–1.22	** < 0.001**
**N-lesions semiquantitative parameters**						
SUVmax at standard imaging	1.33	1.23–1.44	** < 0.001**	1.30	1.12–1.51	** < 0.001**
PSMA score at standard imaging	3.23	2.50–4.18	** < 0.001**	0.55	0.24–1.27	0.16
PSMA RADS at standard imaging	1.93	1.70–2.19	** < 0.001**	1.96	1.51–2.57	** < 0.001**
deltaSUVmax	1.07	1.03–1.11	**0.001**	1.37	1.24–1.53	** < 0.001**

**Fig. 3:**
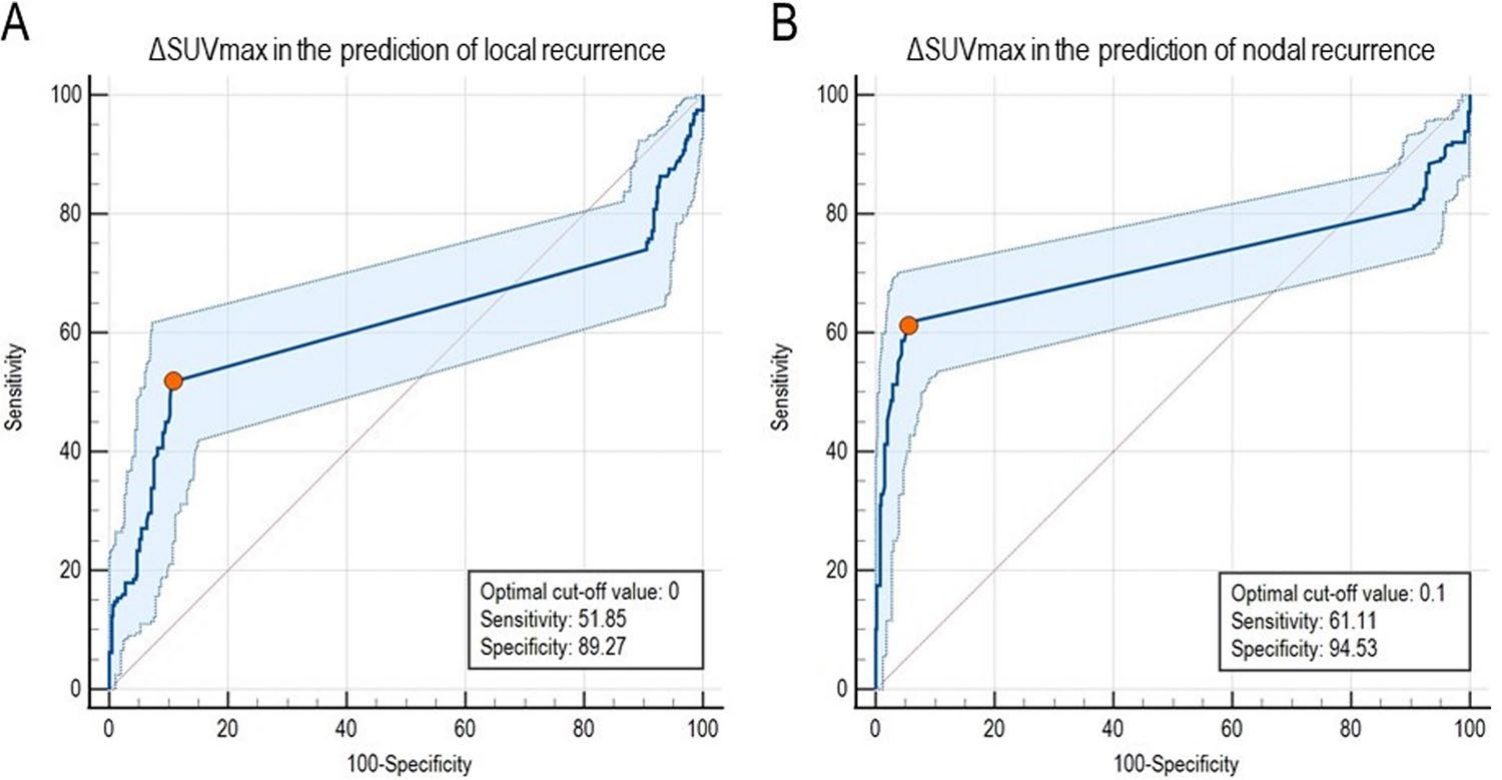
ROC curves of ΔSUVmax in the prediction of local and nodal PCa recurrences. **A**, **B** ROC curves and optimal cutoff values for ΔSUVmax in predicting local and nodal recurrences. The sensitivity and specificity of obtained ΔSUVmax cutoff values are also reported

Optimal ΔSUVmax cutoffs determined by receiver-operating-characteristic AUC analyses to identify local and nodal PCa recurrences were 0 (AUC for cluster data: 0.694; 95% CI: 0.57–0.81) and 0.1 (AUC for cluster data: 0.951; 95% CI: 0.92–0.97), respectively. In other words, even a slight increase or persistence in tracer accumulation at late-phase imaging should be considered suspect for local or nodal PCa recurrences (Fig. 3).

### Impact of forced diuresis late-phase imaging on the interobserver agreement

Adding the forced diuresis late-phase imaging did not improve the interobserver agreement for local recurrences, as it remained moderate at both timepoints (from 80%, kappa = 0.535, 95% CI: 0.42–0.64 to 80%, kappa = 0.524, 95% CI: 0.41–0.63, *p* = ns). By contrast, it increased the agreement from moderate to substantial regarding the nodal assessment (from 80.3%, kappa = 0.585, 95% CI: 0.48–0.68 to 85.9%, kappa = 0.687, 95% CI: 0.59–0.78, *p* < 0.01). Interobserver agreement was not significantly different from standard to standard + forced diuresis late-phase imaging nor for local nor for nodal recurrences once readers were grouped according to the level of expertise (Supplementary Table 2).

## Discussion

Previous studies already showed that adding forced diuresis to the standard [^68^Ga]Ga-PSMA-11 PET/CT protocol improves the visualization of peri-ureteral and peri-bladder tissues by increasing the tracer’s urinary excretion and reducing the so-called halo artifact [8, 10, 11, 22–24]. Late-phase abdominopelvic imaging may further improve the image quality, by prolonging the tracer excretion interval and increasing the signal-to-noise ratio in the prostatic fossa [25–30]. However, it has been shown that delayed imaging can also improve the detection of PCa recurrent sites far from the ureters and bladder [10, 25, 26]. This finding is independent of tracer excretion and might be related to the slower tracer accumulation by low-PSMA-expressing cells, whose visualization might be increased later than 60 min p.i. [10]. Forced diuresis and delayed imaging can thus potentially complement each other rather than being alternative approaches. However, given that furosemide’s half-life is less than 2 h [31], its residual effect may be insufficient to allow for additional late imaging when administered together with or soon after the radiotracer, as currently recommended [10]. Additionally, early furosemide administration may supposedly reduce the tracer’s availability during the uptake phase, resulting in reduced lesion uptake [24, 32]. On these bases, we postponed furosemide administration at 140 p.i. to maximize its pharmacological effect before the late-phase scan [31].

While previous studies tested the added value of modified imaging protocols in terms of detection rate and lesion visibility [27, 29, 32–35], we focused on its impact on diagnostic accuracy by enrolling PET readers with heterogeneous levels of expertise who were asked to follow a standardized PET reporting system [14]. The present results do not support the systematic use of combined forced diuresis late-phase abdominopelvic imaging in addition to the standard scan in the clinical setting, as this protocol slightly (though not significantly) increased diagnostic accuracy. However, this advantage became significant for local uptakes rated by low-experienced readers and for nodal uptakes rated as uncertainly pathological at standard imaging. Low-experienced readers might benefit from the higher visualization of peri-urethral and peri-bladder tissues promoted by the increased tracer excretion. On the other hand, late-phase imaging may better fit the slower tracer kinetics of low-PSMA-expressing cancer cells, thus improving diagnostic accuracy in uncertain nodal uptakes at the standard scan, regardless of the reader’s experience.

**Fig. 4:**
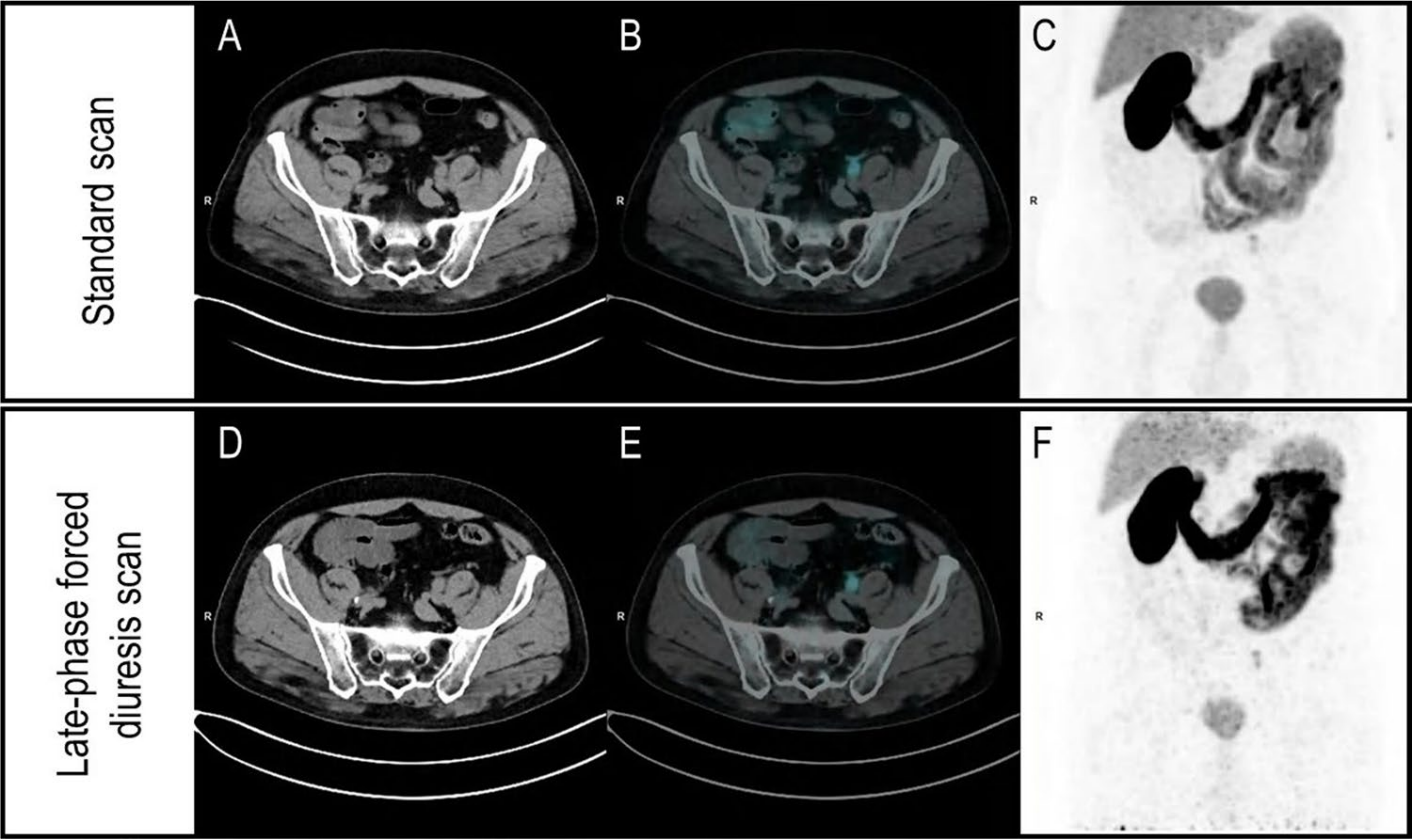
Emblematic example of inconsistent PSMA-RADS and unstructured confidence reporting. The figure displays the case of a 72-year-old patient with a 95% pre-test probability for positive [^68^Ga]Ga-PSMA-11 PET/CT according to [12, 13], showing two equivocal tracer uptakes in soft tissues. (A–C) Axial low-dose CT, fused PET/CT, and whole-body maximum intensity projection images at the standard acquisition. Two areas of equivocal tracer uptake in soft tissues close to the left ureter were present, with upstream dilatated ureter and hydronephrosis in the CT images, consistent with the suspicion of ureteral obstruction. These tracer uptakes were considered equivocal with an unstructured confidence score = 3 and PSMA-RADS 3 by 4/6 PET readers. The same sections represented in panels D–F show that the two areas slightly increased in uptake intensity at the forced diuresis late-phase abdominopelvic scan. After reviewing late-phase images, most PET readers (4/6) judged these two areas suspicious for PCa recurrence, increasing the unstructured confidence score from 3 to 4. By contrast, according to the PSMA-RADS 5-point scale [14], these lesions remained classified as PSMA-RADS 3 in all cases. Given the proximity to the left ureter and the consequent risk of compression, the patient underwent salvage surgery. Post-surgical histopathology confirmed the presence of nodal PCa recurrence

This protocol increased PET readers’ confidence, particularly in lower experienced ones. Notably, confidence improvement was more efficiently tracked using an unstructured five-point scale than PSMA-RADS for both local and nodal lesions. According to the EANM standardized reporting guidelines v1.0, PSMA-RADS frameworks are scored depending on the site and intensity of radiotracer uptake, ranging from very low to very high probability of malignancy [14]. The unstructured confidence assessment had the advantage of greater flexibility allowing PET readers to consider uptake kinetics from early to late-phase imaging, which is not included in PSMA RADS classes. Indeed, only a slight, but not significant, increase in the late-phase PSMA-RADS scores was observed. An emblematic example of inconsistent PSMA-RADS and unstructured confidence reporting is represented in Fig. 4. Notably, dual-phase [^68^Ga]Ga-PSMA-11 PET/CT imaging has been endorsed in the joint EANM/SNMMI guidelines [10] and the inclusion of specific criteria for the interpretation of PSMA uptake kinetics in future updates of PSMA-RADS v1.0 might overcome this limitation.

Interobserver agreement for local restaging was in keeping with previous literature [9, 36] and was not improved by the addition of forced diuresis late-phase to standard imaging. By contrast, the addition of forced diuresis late-phase imaging increased the agreement among readers for the identification of nodal recurrences. In a recent systematic review and meta-analysis, furosemide administration did not favorably impact the interobserver variability for the nodal assessment [36]. However, none of the included studies tested a combination of diuretics and dual-phase imaging.

Coherently with previous data [25, 28, 29, 34, 37, 38], an increase in SUVmax of recurrent PCa lesions was documented on delayed images, suggesting that SUVmax kinetics can distinguish between malignant and benign lesions, as its increase over time is more pronounced in PCa metastases, while its reduction decreases the likelihood of a PCa lesion. In an exploratory analysis, the stability of tracer kinetics identified the optimal cutoff for the presence of local and nodal recurrences (ΔSUVmax = 0, and 0.1, respectively). These cutoffs were not externally validated, and it is generally challenging to translate SUV-derived cutoffs in the clinical setting [39]. Nevertheless, former studies tentatively explored the discriminative potential of SUV-derived cutoffs in [^68^Ga]Ga-PSMA-11 PET/CT, even in multicentric settings [40]. Moreover, ΔSUVmax minimizes the effects of many confounding factors representing the difference between late- and early-phase imaging in the same patient.

The present study has some limitations. First, excluding most healthy organs from the late-phase field of view, we could not directly verify the [^68^Ga]Ga-PSMA-11 in vivo stability. However, this radiopharmaceutical has formerly exhibited excellent stability in several in vitro and in vivo experimental studies [25, 41–43]. Moreover, histopathology was unavailable in most cases. However, a composite reference standard consisting of adequate follow-up imaging and clinical parameters was available for all lesions. Finally, the present results can be applied exclusively for the modified protocol combining forced diuresis and late-phase acquisition in addition to the standard [^68^Ga]Ga-PSMA-11 PET/CT scan. No conclusions can be made regarding the sole forced diuresis addition to the standard scan or delayed imaging without forced diuresis. Similarly, further studies are needed to verify the potential added value of our imaging protocol to other PSMA-targeted radiopharmaceuticals, including urinary excreted ^18^F-fluorinated PSMA (i.e., [^18^F]F-DCFPyL).

In conclusion, a protocol based on standard acquisition followed by combined forced diuresis late-phase imaging is demanding for a nuclear medicine department. The present results do not support its systematic use in the clinical setting but allow the identification of clinical scenarios that might benefit from this protocol. Specifically, it should be considered by low-experienced readers, and when uncertain nodal uptakes are observed at the standard scan.

## Supplementary Information

Below is the link to the electronic supplementary material.

Supplementary file1 (PDF 186 KB)

## Abbreviations

95% CI: 95% Confidence intervals
AUC: Area under the receiver-operating-characteristic curve
GEE: Generalized estimating equation
GS: Gleason Score
ISUP: International Society of Urological Pathology
OR: Odds ratio
PET/CT: Positron emission tomography/computed tomography
PSA: Prostate-specific antigen
PSMA: Prostate-specific membrane antigen
PSMA-RADS: PSMA reporting and data systems
SUVmax: Maximum standardized uptake value

## Declarations

### Guarantor

The scientific guarantor of this publication is Matteo Bauckneht, MD, PhD.

### Statistics and biometry

Alessio Signori has significant statistical expertise.

### Methodology

• prospective

• diagnostic or prognostic study

• performed at one institution
