# Supplementary material for: Combined forced diuresis and late acquisition on [68Ga]Ga-PSMA-11 PET/CT for biochemical recurrent prostate cancer: a clinical practice-oriented study
Source: Eur Radiol. 2023 Mar 9;33(5):3343–53. doi: 10.1007/s00330-023-09516-0 (PMC10121525; doi:10.1007/s00330-023-09516-0)
Supplement: Supplementary file 1 — Supplementary file1 (PDF 186 KB) [file 330_2023_9516_MOESM1_ESM.pdf]

Combined forced diuresis and late acquisition on $[^{68}Ga]$Ga-PSMA-11 PET/CT for biochemical recurrent prostate cancer: a clinical practice-oriented study

Supplementary Materials

1. Supplementary Methods

1.1 Visual, semiquantitative parameters and confidence scores requested from the readers

According to the E-PSMA: the EANM standardised reporting guidelines v1.0 for PSMA-PET [1], nuclear medicine physicians should include both qualitative and quantitative descriptions in their reports to describe PSMA uptake in suspected local (T), nodal (N) or distant prostate cancer (PCa) recurrences (M). Accordingly, in the present study PET readers were asked to indicate the highest maximum standardized uptake value (SUVmax) for the most representative target regions representing the T, N, and M status. In the same sites, the degree of PSMA uptake was asked to be visually compared to reference organs on a visual scale of 0-3, as detailed below:

PSMA visual score 0 = below blood pool
PSMA visual score 1 = equal to or above blood pool and lower than liver
PSMA visual score 2 = equal to or above liver and lower than parotid gland
PSMA visual score 3 = equal to or above parotid gland

For the most representative target regions representing the T, N, and M status, readers were also asked to indicate the degree of confidence, according to the PSMA reporting and data systems (PSMA-RADS) on a scale of 1-5, as detailed below:

PSMA RADS 1 = benign lesion without abnormal PSMA uptake
PSMA RADS 2 = faint PSMA uptake (equal or lower than background) in a site atypical for prostate cancer
PSMA RADS 3 = faint uptake in a site typical for prostate cancer or intense uptake in a site atypical for prostate cancer
PSMA RADS 4 = intense uptake in typical site of prostate cancer, but without definitive findings on CT (A definitive finding on CT means the presence of a real anatomical substrate on the CT)
PSMA RADS 5 = definitive malign lesion

Finally, the readers were asked to assign a personal unstructured confidence score on a scale of 1-5 quantifying the likelihood of the presence of PCa. Compared to PSMA-RADS, when rating this scale the readers were free to assign the likelihood of malignancy regardless of PSMA uptake intensity and the typical or atypical tumour site:

Unstructured confidence score 1 = certain benign
Unstructured confidence score 2 = probably benign
Unstructured confidence score 3 = equivocal
Unstructured confidence score 4 = probably pathological
Unstructured confidence score 5 = certain pathological

2. Supplementary results

2.1 M-status

Regarding the M-status, 7/14 patients (50%) showed multiple lesions, and 6/14 patients (42.8%) had bone metastases. M-recurrences were included in the forced diuresis late acquisition in 9/14 (64.2%) patients. Focusing on true positive M-lesions included in the late scan, SUVmax slightly though not significantly increased (from 10.1±10.5 to 12.5±15.6, p=0.11). Of note, limiting the late-phase scan to the abdominopelvic region, we could not analyse the added value of our imaging protocol regarding the M-status. Considering M-lesions included in the late-phase scan, in contrast to local recurrences and lymph node metastases, only a slight (though not significant) increase in SUVmax was observed. Moreover, previous studies showed that bone or visceral metastases might eventually show no increase or even a decrease

in SUVmax between the early- and the late-phase imaging [2-4]. The analysis of patient cohorts directed to this specific question is still necessary.

3. Supplementary References

[1] Ceci F, Oprea-Lager DE, Emmett L, Adam JA, Bomanji J, Czernin J, et al (2021) E-PSMA: the EANM standardized reporting guidelines v1.0 for PSMA-PET. Eur J Nucl Med Mol Imaging 48:1626-1638.

[2] Alberts I, Sachpekidis C, Gourni E, Boxler S, Gross T, Thalmann G, et al (2020) Dynamic patterns of [68Ga]Ga-PSMA-11 uptake in recurrent prostate cancer lesions. Eur J Nucl Med Mol Imaging 47:160-167.

[3] Morawitz J, Kirchner J, Hertelendy J, Loberg C, Schimmöller L, Dabir M, et al (2022) Is there a diagnostic benefit of late-phase abdomino-pelvic PET/CT after urination as part of whole-body 68 Ga-PSMA-11 PET/CT for restaging patients with biochemical recurrence of prostate cancer after radical prostatectomy? EJNMMI Res 12:12.

[4] C, Kopka K, Eder M, Hadaschik BA, Freitag MT, Pan L, et al (2016) 68Ga-PSMA-11 Dynamic PET/CT Imaging in Primary Prostate Cancer. Clin Nucl Med 41:e473-e479

Supplementary Table 1: Details about general. local. and nodal reports for each reader's category for each round of reporting.

	**standard**						**standard + forced diuresis late-phase**					
	TP	TN	FP	FN	Sens	Spec	TP	TN	FP	FN	Sens	Spec
**General recurrences**												
low experience	83	66	20	31	0.728	0.767	89	71	5	35	0.718	0.934
intermediate experience	77	66	10	47	0.621	0.868	86	62	14	38	0.694	0.816
high experience	67	95	9	29	0.698	0.913	101	67	9	23	0.815	0.882
**Local recurrences**												
low experience	30	123	23	24	0.556	0.842	34	134	12	20	0.630	0.918
intermediate experience	32	123	23	22	0.593	0.842	34	119	26	21	0.618	0.821
high experience	37	123	23	17	0.685	0.842	43	123	23	11	0.796	0.842
**Nodal recurrences**												
low experience	47	109	19	25	0.653	0.852	118	49	10	23	0.837	0.831
intermediate experience	43	112	16	29	0.597	0.875	49	119	9	23	0.681	0.930
high experience	54	105	23	18	0.750	0.820	59	114	14	13	0.819	0.891

FN: false negative; FP: false positive; Sens: sensitivity; Spec: specificity TN: true negative; TP: true positive.

Supplementary Table 2: interobserver agreement for local and nodal recurrences according to the reader's level of expertise

	**standard**			**standard + forced diuresis late-phase**			*p*
	agreement (%)*	*kappa*	*95%CI*	agreement (%)*	*kappa*	*95%CI*	
**Local recurrences**							
low experience	81%	0.552	0.37-0.72	83%	0.548	0.36-0.73	0.71
intermediate experience	69%	0.343	0.19-0.48	70%	0.355	0.20-0.50	0.87
high experience	86%	0.690	0.54-0.83	79%	0.557	0.40-0.70	0.19
**Nodal recurrences**							
low experience	78%	0.540	0.37-0.70	84%	0.638	0.48-0.79	0.28
intermediate experience	76%	0.493	0.33-0.64	80%	0.550	0.38-0.71	0.49
high experience	81%	0.615	0.46-0.76	88%	0.751	0.61-0.88	0.17

* = proportion of consistent reader’s impression
